# 35991 Towards a Novel Robotic Control Scheme to Improve Lower Extremity Movement Post-Stroke

**DOI:** 10.1017/cts.2021.703

**Published:** 2021-03-30

**Authors:** Tom Ruopp, Brian Schmit, Sheila Schindler-Ivens

**Affiliations:** Marquette University

## Abstract

ABSTRACT IMPACT: Effective robotic pedaling therapy would allow stroke survivors a precision, customized, and adaptable therapy to help recover lower extremity function. OBJECTIVES/GOALS: It has been observed that people post-stroke can pedal each limb individually but not simultaneously when the bicycle is split-crank. This implies that lower extremity movement difficulties are affected more by interlimb rather than unilateral coordination deficits. This work seeks to further develop a robotic split-crank pedaling therapy device. METHODS/STUDY POPULATION: This work uses a robotic split-crank pedaling device to facilitate rehabilitation of interlimb coordination, measured by continuous relative phase (CRP) and paretic neuromuscular output. The effects of three control schemes were tested: assist, resist, and assist+resist. Each limb was strapped to a pedal which was connected to a motor. The participants were asked to pedal forward while keeping the pedals antiphase. The robot aided or resisted according to the activated control scheme. Control schemes were tested on two stroke participants. The control schemes respond proportionally to phasing deviation from 180 degrees. The assist scheme assisted the lagging limb while the resist scheme resisted the leading limb. The assist+resist did both control actions. RESULTS/ANTICIPATED RESULTS: For the assist scheme, CRP improved for participant 1 (P1) and declined for participant 2 (P2). P1 increased paretic velocity while P2 decreased. Rectus Femoris (RF) and Biceps Femoris (BF) activity of both limbs lowered for P1. RF and BF activity of both limbs remained about the same but shifted for P2. For the resist scheme, CRP improved for P1 and declined for P2. P1 increased paretic velocity while P2 decreased. P1 increased BF activity of both limbs while RF activity remained constant. P2 increased paretic BF activity and non-paretic RF activity. For the assist+resist scheme, CRP improved for both participants. Both participants increased paretic velocity. P1 increased paretic BF activity and decreased RF activity. P2 better modulated paretic BF and RF. DISCUSSION/SIGNIFICANCE OF FINDINGS: All control schemes augmented performance, however the assist+resist scheme showed the most promise in terms of CRP and muscle activity. More participants are needed to determine true effects of each control scheme. The control scheme selected will be the foundation for further improvements such as adaptive control and extrinsic feedback.

